# Pharmacokinetics, bioavailability and dose assessment of Cefquinome against *Escherichia coli* in black swans (*Cygnus atratus*)

**DOI:** 10.1186/s12917-017-1148-7

**Published:** 2017-07-28

**Authors:** Dong-Hao Zhao, Xu-Feng Wang, Qiang Wang, Liu-Dong Li

**Affiliations:** 1 Key Laboratory of Aquatic Product Processing, Ministry of Agriculture, Guangzhou, People’s Republic of China; 2 Laboratory of Quality & Safety Risky Assessment for Aquatic Product on Storage and Preservation, Guangzhou, People’s Republic of China; 3 Fishery Environments and Aquatic Products Quality Inspection & Testing Center of the Ministry of Agriculture, Guangzhou, People’s Republic of China; 4 South China Sea Fisheries Research Institute, Chinese Academy of Fishery Sciences, Guangzhou, 510300 People’s Republic of China

**Keywords:** Cefquinome, Black swans, Pharmacokinetics, Monte Carlo analysis

## Abstract

**Background:**

The objective of this study is to investigate pharmacokinetics and dose regimens of cefquinome in black swans following intravenous (IV) and intramuscular (IM) administration at a single dose of 2 mg/kg. The MICs of cefquinome against 49 *Escherichia coli* isolates from black swans were determined. Monte Carlo simulation was applied to conduct the dose regimen assessment and optimization of cefquinome against *E. coli* in black swans, and a pharmacokinetic/pharmacodynamic (PK/PD) cutoff was established for *E. coli* isolates obtained in this study.

**Results:**

The PK parameters of T_1/2α_ (0.31 h), T_1/2β_ (1.69 h) and Cl_B_ (0.13 L/kg·h) indicated a rapid distribution and elimination of cefquinome in black swans after IV administration. After IM injection, the corresponding PK parameters of T_1/2Ka_, T_1/2Ke_, T_max_, C_max_, and F were 0.12 h, 1.62 h, 0.39 h, 5.71 μg/mL and 74.2%, respectively. The MICs of cefquinome against black swans *E. coli* ranged from 0.03 to 8 μg/mL, with MIC_50_ and MIC_90_ of 0.06 and 0.5 μg/mL, respectively. The PK/PD cutoff of cefquinome against *E. coli* was determined to be 0.2 μg/mL. Monte Carlo simulation showed that the nominal dose regimen (2 mg/kg/24 h) could not achieve a satisfactory probability of target attainment (PTA) for %T_MIC_ ≥ 50%, indicating a risk of treatment failure and the development of potential drug resistance.

**Conclusions:**

The current daily dosage of cefquinome when divided into 12-h interval (1 mg/kg/12 h) may be effective for the treatment of *E. coli* infections with an MIC ≤0.5 μg/mL.

**Electronic supplementary material:**

The online version of this article (doi:10.1186/s12917-017-1148-7) contains supplementary material, which is available to authorized users.

## Background

The black swan (*Cygnus atratus*) is a large black-feathered waterbird which breeds mainly in New Zealand, Australia and adjacent coastal islands with nomadic migration patterns dependent on climatic conditions. As a popular ornamental bird, black swans have been introduced into numerous countries and have formed stable populations in zoological gardens [1]. In general, clinical bacterial infections caused by *Enterobacteriaceae* are common in waterfowl [2]. Additionally, *Escherichia coli*, *Salmonella* spp. and *Campylobacter* spp. infections can have serious detrimental on waterfowl populations and pose a potential threat to human public health as well. A recent study has reported the emergence of carbapenem and colistin-resistant *E.coli* isolates co-carrying *bla*_NDM-5_ and *mcr-1* genes in the fowls [3], indicating the possible spread and prevalence of such resistant strains through the food chain and migration of wild birds.

Cefquinome is the 4th generation cephalosporin antibiotic developed solely for veterinary use and has been approved for the treatment of many diseases including respiratory tract disease, foot rot in cattle, calf septicemia, metritis-mastitis-agalactia syndrome in sows, foal septicemia and respiratory diseases in horses [4]. Cefquinome is routinely used at a single dose of 2 mg/kg BW once-daily [5]. The advantages of cefquinome include broad-spectrum antimicrobial activity, highly stable against β-lactamases, enhanced potency and the ability to penetrate easily into Gram-negative bacterium [6]. Previous pharmacokinetic studies of cefquinome have been conducted in various species including pigs, cattle, beagle dogs, wild boars and ducks [7–11]. However, no relavant study regarding administration of cefquinome in wild animals was reported, and most previous studies were focused on the characteristics of drug disposition without regard to the appropriate dose regimen assessment for therapeutic use of cefquinome. Here we report, to the best of our knowledge, for the first time cefquinome PK properties and dose regimens assessment in wild birds.

In the present study, we present the PK profile and bioavailability of cefquinome in black swans. The MICs of cefquinome against 49 *E. coli* isolates from black swans were also determined. In addition, Monte Carlo analysis was performed to derive the corresponding daily dose regimens required to achieve the specific activity for various MIC breakpoints based on the determined PK parameters in black swans, the MIC distribution and the reported PD targets [12]. These results may provide fundamental data for the assessment of clinical efficacy of cefquinome and suggestions for a more rational dose regimen in black swans expected of having *E. coli* infections.

## Methods

### Animals and experimental design

Twelve healthy black swans weighting 5.14 ± 0.79 kg were divided equally into two groups. Each group randomly included three males and three females. Swans in each group received cefquinome (cefquinome sulfate injection, 40 mg/mL, Qilu Animal Health Products Co., Ltd., Jinan, China) at a single dose of 2 mg/kg/24 h BW by IV (brachial vein) or IM (chest muscle) injection. The black swans used in this study were kindly provided by the Lv-Yuan Rare Bird Farm (Shangdong, China). All birds were raised in accordance with the National Standards for Laboratory Animals of China (GB 14925–2010), allowed ad libitum access to water and antibacterial-free feedstuffs. The animal experimental protocol was approved by the Committee on the Ethics of animals of South China Sea Fisheries Research Institute and the Institutional Animal Care and Use Committee of Lv-Yuan Rare Bird Farm.

### Sample collection and analysis

Blood (0.5 mL) was collected from the contralateral brachial vein with heparin sodium before and at 0.083, 0.25, 0.5, 1, 2, 3, 4, 6, 8, 12 and 24 h following IV or IM administration. Plasma samples were then immediately isolated by centrifugation at 4000 rpm for 10 min and stored at −80 °C until further analysis.

A 0.2 mL aliquot of plasma sample was transferred into a capped centrifuge tube, and then mixed with 0.2 mL of acetonitrile. After vortexing (30 s) and centrifuging (12,000 rpm, 10 min), the supernatant was filtered through a 0.22 μm nylon syringe filter and collected into a sample vial for concentration determination. The plasma cefquinome concentrations were determined using a modified high performance liquid chromatography tandem mass spectrometry (HPLC-MS/MS) method as our previously reported [9] (details are given in Additional file 1). The calibration standards in the linear range of 0.01–0.5 μg/mL were produced by working solutions spiked in blank plasma after extraction. All samples that had concentrations above 0.5 μg/mL were diluted proportionally with the control plasma prior to extraction with acetonitrile. The limit of detection (LOD) and quantification (LOQ) for cefquinome in plasma were set according to signal-to-noise (S/N) ratio of 3:1 and 10:1, respectively. The analytical method was validated by assessing extraction efficiency and inter- and intra-day reproducibility at drug concentrations of 0.01, 0.1 and 0.5 μg/mL.

### Pharmacokinetic analysis

Pharmacokinetic parameters of cefquinome were estimated by a compartmental method using WinNonlin software (version 6.1, Pharsight, St. Louis, MO, USA). The best-fitting model required to describe cefquinome time-concentration curves for each swan was determined by application of the weighed residual sums of square and Akaike Information Criterion (AIC) methods [13]. The time-concentration of IV route was best fitted in WinNonlin program using a two-compartment model as presented in Model 7: Concentration (T) = Ae^-α∙T^ + Be^-β∙T^. The IM route was best fitted in WinNonlin program using a one-compartment model with the first-order absorption as presented in Model 3: $\mathrm{Concentration}\ \left(\mathrm{T}\right)=\frac{\mathrm{Dose}\kern0.75em \cdot \kern0.5em Ka}{{\mathrm{V}}_{\mathrm{d}}\ \left(Ka\hbox{-} Ke\right)}\ {\mathrm{e}}^{-Ke\cdot \mathrm{T}}\hbox{-} {e}^{-Ka\cdot \mathrm{T}}$. For IV dosing, the distribution and elimination half-lives were estimated as T_1/2α_ = 0.693/α and T_1/2β_ = 0.693/β, respectively. The half-lives of the first-order absorption and elimination after IM injection were correspondingly calculated as T_1/2Ka_ = 0.693/Ka and T_1/2Ke_ = 0.693/Ke. The other PK parameters, peak plasma concentration (C_max_), the time to C_max_ (T_max_), the apparent steady-state volume of distribution (V_ss_), total area under time-concentration curve (AUC) and total body clearance (Cl_B_) were also calculated in WinNonlin software. The bioavailability (F%) was calculated according to the standard equations as follows [14]: $\mathrm{F}=\kern0.75em \frac{AUC_{IM}}{AUC_{IV}}\times 100\%$. All PK parameters were presented as mean ± SD values.

### MIC determination

A total of 49 *E. coli* strains were obtained from more than 300 faecal swabs of black swans breeded in five different separated populations between 2014 and 2015. The MICs of cefquinome for these *E. coli* isolates were determined using the standard CLSI microdilution method [15]. The MICs for 50% and 90% of the isolates (MIC_50_ and MIC_90_, respectively) were calculated accordingly.

### Monte Carlo analysis and dose assessment

For β-lactam antibiotics acting by the time-dependent killing mechanisms, it is commonly recommended that the duration of time that drug levels exceed the MIC (%T_MIC_) should be at least 50% and possibly more than 80% of the dosing interval to ensure an appropriate bactericidal effect [12]. To further assess the recommended dose regimens of cefquinome in black swans against *E. coli*, a 10,000-subject Monte Carlo analysis was conducted using the Crystal Ball Professional software (version 7.2.2; Oracle Corporation), based on the current PK parameters, MIC distribution and the PD targets (%T_MIC_ > 50 or 80%).

The %T_MIC_ values after IM injection was calculated using the following equation: $\mathrm{Concentration}\kern0.75em \left(\mathrm{t}\right)\kern0.5em =\kern0.5em \frac{\mathrm{Dose}\kern0.75em \cdot \kern0.5em Ka\kern0.75em \cdot \mathrm{F}}{{\mathrm{V}}_{\mathrm{d}}\ \left(Ka\hbox{-} Kel\right)}\ {\mathrm{e}}^{\hbox{-} Ke\cdot \mathrm{t}}\hbox{-} {\mathrm{e}}^{\hbox{-} Ka\cdot \mathrm{t}}$, where concentration is the MIC, V_d_ is the apparent volume of distribution, F is bioavailability, Ka is constant of absorption rate and Ke is constant of elimination rate for IM administration. All PK parameters were assumed to be normally distributed in the form of mean and standard deviation (Table 1). In order to obtain a unimodal distribution, the two isolates with a MIC of 8 μg/mL were consequently removed, and the log_2_-transformed MIC distribution of the other 47 isolates was submitted to the non-linear least squares regression and standard goodness-of-fit test. The probability of attaining the %T_MIC_ targets at the specific MICs was accordingly calculated. The PK/PD cutoff (CO_PD_) is defined as the MIC, at which the PTA for %T_MIC_ target (50%) was equal to 90% under the current clinical recommended dose (2 mg/kg/24 h). For calculation of daily dose regimens, the MIC was defined as a single value ranging from 0.03 to 8 μg/mL. Scenarios were simulated separately for a single IM injection of cefquinome from 0 to 265 mg/kg with 24-h or 12-h intervals. The precise recipes of cefquinome required to achieve the specific activity (%T_MIC_ > 50 or 80%) against *E. coli* isolate at each MIC in black swans were also estimated based on PK data, Monte Carlo analysis and the equation mentioned above.

**Table 1:** Mean ± SD values of the pharmacokinetic parameters of cefquinome in black swans after IV and IM administration at a dose of 2 mg/kg BW

Parameter	Unit	IV	IM
Ka	1/h	—	5.64 ± 3.07
A	μg/mL	10.7 ± 4.97	—
α	1/h	2.27 ± 0.23	—
Kel	1/h	—	0.43 ± 0.03
B	μg/mL	4.20 ± 1.91	—
β	1/h	0.42 ± 0.09	—
V_ss_	L/kg	0.32 ± 0.17	—
T_1/2Ka_	h	—	0.12 ± 0.04
T_1/2α_	h	0.31 ± 0.03	—
T_1/2Ke_	h	—	1.62 ± 0.11
T_1/2β_	h	1.69 ± 0.85	—
T_max_	h	—	0.39 ± 0.19
C_max_	μg/mL	—	5.71 ± 1.43
AUC	μg·h/mL	16.5 ± 4.92	12.17 ± 4.32
Cl_B_	L/kg·h	0.13 ± 0.04	—
F	%	—	74.2 ± 26.3

A, zero-time intercept of the distribution slope in the compartment model; B, zero-time inter of decline in plasma concentration of drug; α, distribution rate constant; β, elimination constant; Ke, constant of elimination rate; Ka, constant of absorption rate; T_1/2Kel_, elimination half-life; T_1/2α_, the distribution half-life; T_1/2β_, the half-life of elimination; T_1/2Ka_, absorption half-life; V_ss_, the apparent steady-state volume of distribution; Cl_B_, total body clearance; AUC, total area under the concentration–time curve from zero to infinity; T_max_, time to C_max_ from time zero; C_max_, peak plasma concentration; F, bioavailability

## Results

### Cefquinome concentration assay in swan plasma

The matrix-matched calibration curve was linear between 0.01 and 0.5 μg/mL, with a coefficient of determination (R^2^) of 0.998. The LOQ and LOD were 0.01 and 0.005 μg/mL, respectively. Mean extraction recoveries from the five replicate assays were 88.9 ± 7.96%, 95.2 ± 7.20% and 98.3 ± 7.97% at spiked drug concentrations of 0.01, 0.1, and 0.5 μg/mL, respectively. The intraday coefficients of variation for replicate control samples (*n* = 5) within these concentration ranges varied from 1.52 to 5.69%, and the interday coefficients of variation ranged from 5.96 to 7.18%.

### Pharmacokinetics of cefquinome in plasma

**Fig. 1:**
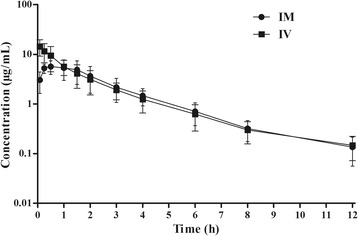
Semi-logarithmic plot of plasma concentration-time profiles of cefquinome after IV and IM administration at a single dose of 2 mg/kg BW. Values are mean ± SD (*n* = 6)

No adverse effect or intolerance was observed during the entire experiment. The plasma time-concentration profiles are plotted in Fig. 1, and the corresponding PK parameters are summarized in Table 1. Cefquinome exhibited a biphasic decline and showed the best fit to a two-compartmental open model after IV dosing in black swans. The distribution half-life (T_1/2α_) and elimination half-life (T_1/2β_) were 0.31 ± 0.03 h and 1.69 ± 0.85 h, respectively. After IM injection, the drug time-concentration data was best described by a one-compartment model with first-order absorption. Cefquinome was absorbed rapidly with an absorption half-life (T_1/2Ka_) of 0.12 h. The peak concentration (C_max_; 5.71 μg/mL) was achieved at 0.39 h, and the bioavailability was 74.2 ± 26.3% after IM injection. No drug was detected at 24 h after cefquinome administration.

### MIC distribution

**Fig. 2:**
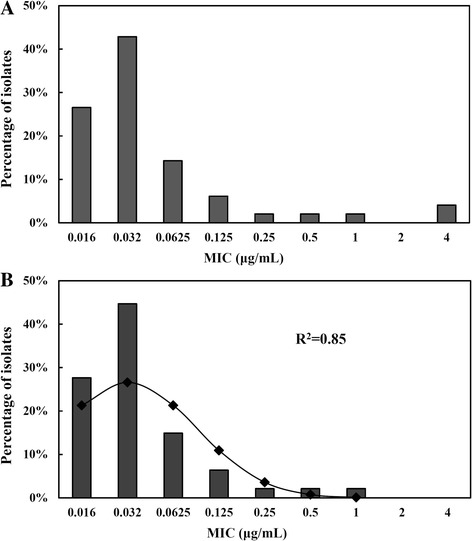
The MIC distribution of cefquinome against *E. coli* isolates from black swans. **a** Primary MIC distribution of 49 *E. coli* isolates; (**b**) Fitted MIC distribution of the estimated 47 *E. coli* isolates after the goodness-of-fit tests and nonlinear least-squares regression. The *lines* indicate fitted theoretical normal distribution values

As shown in the primitive cefquinome MIC distribution in Fig. 2a, the MICs for cefquinome against 49 *E. coli* strains isolated from black swans were in the range of 0.03 to 8 μg/mL. The MIC_50_ and MIC_90_ were determined to be 0.063 and 0.5 μg/mL, respectively (Additional file 2: Table S1). The distribution percentage at each MIC (0.03, 0.06, 0.13, 0.25, 0.5, 1, 2 and 8 μg/mL) was 26.5%, 42.9%, 14.3%, 6.1%, 2.0%, 2.0%, 2.0% and 4.1%, respectively. In the fitted log_2_-transformed MIC distribution of the 47 isolates, the best fit for the unimodal population was found (*R* ^*2*^ = 0.85) when presumed MIC distribution was defined as being between 0.03 and 2.0 μg/mL.

### Monte Carlo analysis and dose assessment

**Fig. 3:**
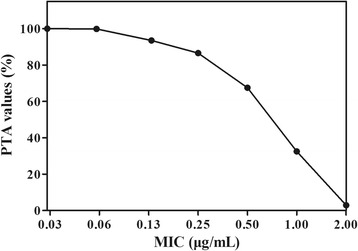
The probability of target attainment (PTA) of cefquinome treated with the current clinical dose regimen (2 mg/kg/24 h) against *E. coli* at each MIC value

**Fig. 4:**
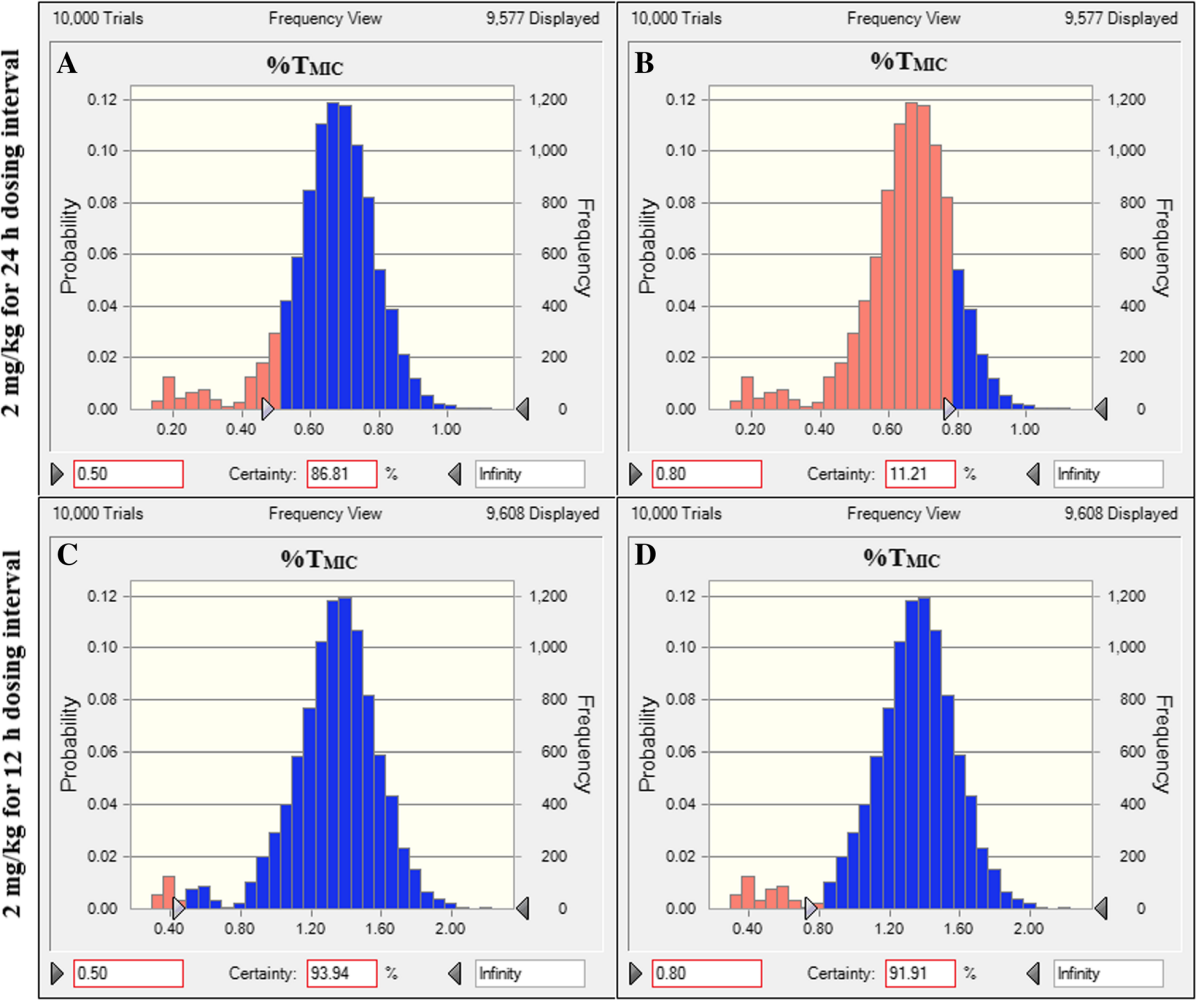
The probability distribution of the calculated %T_MIC_ for cefquinome using a 10,000-subject Monte Carlo analysis based on the measured PK parameters obtained following IM injection at 2 mg/kg BW with 24-h (**a** and **b**) and 12-h (**c** and **d**) dosing interval in black swans and *E. coli* MIC distribution in this study. The areas of blue columns represent the probability of target attainment (PTA) for %T_MIC_ ≥ 50 or 80%

The PTA values of cefquinome administered at single dose of 2 mg/kg/24 h against *E. coli* at each MIC were presented in Fig. 3. When MIC of *E. coli* was below 0.2 μg/mL, the PTA for achieving %T_MIC_ > 50% was as high as 90.2%. Therefore, the PK/PD cutoff of cefquinome against 49 *E. coli* isolates obtained in our study was 0.2 μg/mL. In addition, the calculated daily dosages of cefquinome required to achieve the specific activity (%T_MIC_ > 50% or 80%) against *E. coli* isolates with different MICs in black swans were summarized in Table 2. A simulated dose regimen (1.86 mg/kg/24 h) would be only therapeutically effective against *E. coli* with MIC ≤0.125 μg/mL in black swans. However, the similar daily total dose (1.94 mg/kg) may achieve a successful therapy for *E. coli* with a MIC of ≤0.5 μg/mL after splitting the dose into 12-h intervals (0.97 mg/kg/12 h). Similarly, as shown in Fig. 4, for the used *E. coli* MIC distribution in the present study, a PTA of 11.2% for %T_MIC_ ≥ 80% was acquired when cefquinome administered at 2 mg/kg once-daily, while 91.9% could be achieved if the same dose given at 12-h intervals (1 mg/kg/12 h).

**Table 2:** Calculated total daily dose of cefquinome required to achieve the specific activity (%T_MIC_ > 50% or 80%) against *E. coli* isolates from black swans with diverse MIC values

MIC (μg/mL) for *E. coli* isolates	Total daily dose (mg/kg) of cefquinome required to achieve			
	%T_MIC_ > 50%		%T_MIC_ > 80%	
	24-h interval	12-h intervals	24-h interval	12-h intervals
0.031	0.45	0.14	3.88	0.38
0.063	0.89	0.24	7.95	0.72
0.125	1.86	0.48	16.4	1.68
0.25	3.25	0.98	33.7	2.96
0.5	7.38	1.94	67.3	6.54
1	14.6	3.86	132.7	12.9
2	29.7	7.72	264.2	27.4
8	115.1	31.2	−	106.6

## Discussion

This study was the first of its kind to investigate PK of cefquinome applied in wild birds. The elimination half-life (T_1/2β_) of cefquinome after IV administration was 1.69 h, which was similar to those values reported in chickens (1.29 h) and ducks (1.57 h), indicating a relatively short drug persistence in black swans [11, 16]. However, an evidently shorter elimination half-life was observed in beagle dogs (0.98 h) or sheep (0.78 h) [9, 17]. Similarly, a more rapid absorption (T_1/2Ka_) of cefquinome after IM injection were acquired in black swans (0.12 h), chickens (0.07 h) and ducks (0.12 h) compared with in mammals such as piglets (0.41 h), sheep (0.31 h) and camels (4.35 h) [11, 17–20]. This PK property represented a shorter duration for the drug to reach systemic circulation and to more rapidly establish of effective drug concentration in birds.

The diffusion of cefquinome in the body tissues of different animal species is very small. In this study, barely 0.32 L ⁄kg of V_ss_ was obtained in black swans similar to 0.25 L/kg in piglets and 0.19 L/kg in pigs [21, 22]. According to the CVMP report, an attenuated distribution of cefquinome to intracellular spaces was due to low fat solubility and that it acts as an organic acid with a low pKa of 2.51 or 2.91 [5]. In addition, total body clearance (Cl_B_) in black swans was determined to be 0.13 ± 0.04 L/kg·h in this study, which is similar to 0.18 L/kg in rabbits or 0.12 L/kg in horse but lower than the corresponding value of 0.26 L/kg in piglets [18, 23, 24].

As an animal-specific cephalosporin, cefquinome has been widely employed in veterinary medicine due to excellent antimicrobial activity. Cefquinome is considered to be a time-dependent antimicrobial agent, and %T_MIC_ is the dominant PK/PD index correlated with the therapeutic efficacy [25]. The previous PK/PD studies in animal infection models have demonstrated that drug levels of β-lactam antibiotics needed to exceed the MIC for 36% to 40% of dosing interval to exert an in vivo bacteriostatic effect against *Enterobacteriaceae* [26]. In general, the magnitude of %T_MIC_ to ensure a significant bactericidal or virtual elimination effect should be at least 50% of the recommended dosing interval [12, 27].

The PK/PD cutoff is crucial for guiding clinical use of antimicrobials. For most β-lactam antibiotics, the CLSI and EUCAST have established the CO_PD_ from human studies. However, as an important index reflecting the variations in host species PK and bacterial species MIC distribution, the CO_PD_ is normally significantly different between human and animals [28]. Currently, no breakpoint data of cefquinome was established for animal infections caused by *E. coli*. In the present study, the CO_PD_ of cefquinome against *E. coli* in black swans was determined to be 0.2 μg/mL at the recommended dose (2 mg/kg/24 h) based on Monte Carlo analysis, which was lower than the EUCAST clinical CO_PD_ values of cefotaxime (2 μg/mL), cefpodoxime (1 μg/mL), ceftriaxone (2 μg/mL), cefixime (1 μg/mL) and ceftibuten (1 μg/mL) against *Enterobacteriaceae* [29]. However, as only 49 *E. coli* isolates were used in our study due to the limited population of black swans, the relatively conservative CO_PD_ should be verified in a larger number of bacteria and clinical practices.

For dosage regimen assessment, Monte Carlo analysis is an important computing tool that is useful to predict the attainment of therapeutic efficacy and determine the CO_PD_ according to PK data, MIC distribution and the magnitude of PK/PD indices. As seen in Table 2, the increasing total amount of drug exerted little added therapeutic efficacy even with a considerably high simulated dose. However, after splitting into 12-h intervals, the identical daily dose could achieve a more satisfactory outcome than a single dosing. Therefore, more frequent administrations are needed for cefquinome to obtain a longer treatment period in the form of %T_MIC_. Routinely, a twice-daily schedule is considered a good compliance target in the clinical practice. Based on the current PK study, MIC distribution and specific PD targets, if the dose is given at 1 mg/kg twice daily, the 10,000-subject Monte Carlo simulation showed that the PTA of 95.9% and 89.2% could be achieved for %T_MIC_ > 50% and 80% targets, respectively, against *E. coli* isolates in this study. In addition, taking into account the recently reported %T_MIC_ target of 51.7% required to achieve a 2-log_10_ killing effect in murine thigh infection against *E. coli* isolates [27], cefquinome 1 mg/kg/12 h is estimated to be effective against *E. coli* infection in black swans.

## Conclusions

To our knowledge, it is the first report about the PK and dose assessment study for cefquinome in black swans targeting *E. coli* strains. In the present study, we investigated pharmacokinetics and bioavailability of cefquinome following IV and IM administration in black swans. The MICs of cefquinome against 49 *E. coli* isolates from black swan were also determined. On the basis of PK data, MIC distribution and PD analysis, this study evaluated dose regimens of cefquinome in black swans against *E. coli* infections. Our findings suggest that the daily dose regimen of cefquinome at 1 mg/kg/12 h would be appropriate to achieve a satisfactory efficacy in the treatment of infections caused by *E. coli* in black swans.

## Additional files

Additional file 1: LC-MS/MS method. (PDF 42 kb)

Additional file 2: Table S1. MICs of cefquinome (μg/mL) against *E. coli* strains isolated from black swans. (PDF 47 kb)

## Abbreviations

%T_MIC_: The duration of time that drug levels exceed the MIC in dosing interval
BW: Bodyweight
CLSI: Clinical and laboratory standards institute
EUCAST: European committee on antimicrobial susceptibility testing
IM: Intramuscular
IV: Intravenous
MIC: Minimal inhibitory concentration
PD: Pharmacodynamic
PK: Pharmacokinetic
PTA: Probability of target attainment
