# Supplementary material for: Pharmacokinetics, bioavailability and dose assessment of Cefquinome against Escherichia coli in black swans (Cygnus atratus)
Source: BMC Vet Res. 2017 Jul 28;13:226. doi: 10.1186/s12917-017-1148-7 (PMC5534040; doi:10.1186/s12917-017-1148-7)
Supplement: Supplementary file 1 — LC-MS/MS method. (PDF 42 kb) [file 12917_2017_1148_MOESM1_ESM.pdf]

Supplementary Materials & Methods

LC-MS/MS method

The drug concentration analyses were performed using a HPLC-ESI-MS/MS system (Agilent 1200 HPLC system; Agilent Technologies, Santa Clara, CA, USA; API 4000 triple quadrupole mass spectrometer; Applied Biosystems, Carlsbad, CA, USA) equipped with a short column (Waters Symmetry C18, 2.1×50 mm, 3.5 μm). The injection volume was 5 μL, and column temperature should be maintained at 35 °C. The mobile phase consisted of acetonitrile (A) and 0.1% formic acid in water containing 2 mM ammonium acetate (B) using a gradient elution with a flow rate of 200 μL/min: 0.0-1.5 min (5-60% A), 1.5-4.5 min (60% A), 4.5-5.5 min (60-5% A), 5.5-11 min (5% A). The total run time was 11 min. The mass conditions were as follows: ionspray voltage, 4000 V; curtain gas, 20 psi; nebulizer gas, 55 psi; collision gas, 20 psi; source temperature, 500 °C. Ion transitions of m/z 529.3→134.1 and 529.3→396.2 were chosen for multiple reaction monitoring (MRM) experiments in positive mode.
