# Supplementary material for: Pharmacokinetics, bioavailability and dose assessment of Cefquinome against Escherichia coli in black swans (Cygnus atratus)
Source: BMC Vet Res. 2017 Jul 28;13:226. doi: 10.1186/s12917-017-1148-7 (PMC5534040; doi:10.1186/s12917-017-1148-7)
Supplement: Supplementary file 2 — MICs of cefquinome (μg/mL) against E. coli strains isolated from black swans. (PDF 47 kb) [file 12917_2017_1148_MOESM2_ESM.pdf]

**Table S1.** MICs of cefquinome (μg/mL) against *E.coli* strains isolated from black swans.

| Number | MIC | Number | MIC | Number | MIC | Number | MIC | Number | MIC | Number | MIC | Number | MIC |
|---|---|---|---|---|---|---|---|---|---|---|---|---|---|
| Z1D1 | 0.03125 | Z1D8 | 0.0625 | Z2D16 | 0.25 | Z2D8 | 0.0625 | Z3D16 | 0.0625 | Z3D5 | 0.03125 | Z3P3 | 0.03125 |
| Z1D2 | 0.03125 | Z1D9 | 0.25 | Z2D2 | 1.0 | Z2D9 | 0.0625 | Z3D17 | 0.0625 | Z3D6 | 8.0 | Z3P4 | 0.5 |
| Z1D3 | 0.03125 | Z2D1 | 0.03125 | Z2D3 | 0.25 | Z3D1 | 0.125 | Z3D18 | 0.0625 | Z3D7 | 0.0625 | Z3P5 | 0.03125 |
| Z1D4 | 0.03125 | Z2D10 | 0.0625 | Z2D4 | 0.0625 | Z3D10 | 0.125 | Z3D2 | 0.0625 | Z3D8 | 0.125 | Z3P6 | 0.03125 |
| Z1D5 | 0.03125 | Z2D11 | 0.0625 | Z2D5 | 0.0625 | Z3D11 | 0.0625 | Z3D3 | 0.0625 | Z3D9 | 0.125 | Z3P7 | 0.0625 |
| Z1D6 | 0.03125 | Z2D13 | 0.0625 | Z2D6 | 2.0 | Z3D12 | 0.125 | Z3D4 | 0.125 | Z3P1 | 8.0 | Z3P8 | 0.0625 |
| Z1D7 | 0.03125 | Z2D14 | 0.0625 | Z2D7 | 0.0625 | Z3D15 | 0.0625 | Z3D16 | 0.0625 | Z3P10 | 0.0625 | Z3P9 | 0.125 |
